# Dynamic Nucleosome Organization at *hox* Promoters during Zebrafish Embryogenesis

**DOI:** 10.1371/journal.pone.0063175

**Published:** 2013-05-09

**Authors:** Steven E. Weicksel, Jia Xu, Charles G. Sagerström

**Affiliations:** 1 Department of Biochemistry and Molecular Pharmacology, University of Massachusetts Medical School, Worcester, Massachusetts, United States of America; 2 Bioinformatics Core, University of Massachusetts Medical School, Worcester, Massachusetts, United States of America; Radboud University Nijmegen, The Netherlands

## Abstract

Nucleosome organization at promoter regions plays an important role in regulating gene activity. Genome-wide studies in yeast, flies, worms, mammalian embryonic stem cells and transformed cell lines have found well-positioned nucleosomes flanking a nucleosome depleted region (NDR) at transcription start sites. This nucleosome arrangement depends on DNA sequence (cis-elements) as well as DNA binding factors and ATP-dependent chromatin modifiers (trans-factors). However, little is understood about how the nascent embryonic genome positions nucleosomes during development. This is particularly intriguing since the embryonic genome must undergo a broad reprogramming event upon fusion of sperm and oocyte. Using four stages of early embryonic zebrafish development, we map nucleosome positions at the promoter region of 37 zebrafish *hox* genes. We find that nucleosome arrangement at the *hox* promoters is a progressive process that takes place over several stages. At stages immediately after fertilization, nucleosomes appear to be largely disordered at *hox* promoter regions. At stages after activation of the embryonic genome, nucleosomes are detectable at *hox* promoters, with positions becoming more uniform and more highly occupied. Since the genomic sequence is invariant during embryogenesis, this progressive change in nucleosome arrangement suggests that trans-factors play an important role in organizing nucleosomes during embryogenesis. Separating *hox* genes into expressed and non-expressed groups shows that expressed promoters have better positioned and occupied nucleosomes, as well as distinct NDRs, than non-expressed promoters. Finally, by blocking the retinoic acid-signaling pathway, we disrupt early *hox* gene transcription, but observe no effect on nucleosome positions, suggesting that active *hox* transcription is not a driving force behind the arrangement of nucleosomes at the promoters of *hox* genes during early development.

## Introduction

The nucleosome is comprised of an octamer histone core wrapped nearly 1.7 times by approximately 147 bp of DNA that represents the basic unit of eukaryotic chromatin [1]. While packaging of nucleosomes into a higher order structure enables the compaction of chromatin into the nucleus, it also limits access to various DNA binding factors, thereby placing an accessibility constraint on all DNA-dependent processes (e.g. replication, transcription) [2]. Nucleosome arrangements on genomic DNA are defined both in terms of positioning (how precisely a nucleosome resides at a particular site in all cells of a population) and occupancy (how frequently a specific position is bound by a nucleosome). In particular, nucleosome positioning and occupancy at transcription start sites (TSSs) is thought to impact gene expression. Accordingly, genome-wide nucleosome mapping studies in yeast have revealed a nucleosome-depleted region (NDR) upstream of most TSSs [3]–[7] that likely permits access by the transcription machinery. However, some yeast promoters appear to be occupied by nucleosomes that are actively removed in response to inducing signals [8]–[10]. Such promoters display higher transcriptional plasticity and are more responsive to signaling pathways, than are promoters with pronounced NDRs, suggesting that nucleosome positioning represents a mechanism to achieve regulated gene expression in yeast [11]. Nucleosome positioning may play an even greater role in the regulation of gene expression in metazoans since regulatory DNA sequences are invariant among all cells of a multi-cellular organism, but only a subset of cells may express a specific gene. Indeed, while many promoters in flies [12]–[14], worms [15], [16], fish [17], and humans [18], [19] display NDRs upstream of TSSs, many other promoters are occupied by nucleosomes [20] and inductive signals cause nucleosome rearrangements at such promoters (e.g. nucleosome occupancy is greatly increased in the region immediately upstream of repressed promoters upon T-lymphocyte stimulation [19] and NDRs form at androgen-responsive enhancers in prostate cells [21]). This suggests that nucleosomes need to be rearranged at many metazoan promoters prior to transcription and, accordingly, there is an overall bias towards expressed promoters having a more pronounced NDR [12], [18], [19].

Nucleosome positioning is partially encoded by the DNA sequence and experimental studies have identified sequences that favor (e.g. dinucleotide repeats [22], [23] and G+C rich regions [5], [24]) or disfavor (e.g. dA:dT tracts [3], [5], [18], [25], [26]) nucleosome binding. More recently, experimentally derived nucleosome position information has been used to design theoretical models for the purpose of predicting nucleosome positioning *de novo*. These models are reasonably successful at predicting nucleosome positions in yeast [24], [27]–[29], but are less successful in *C. elegans* [7] or in human cells [20]. In particular, the models appear less accurate at predicting nucleosome positioning at metazoan regulatory regions (including promoters [7], [20]). Notably, regulatory regions have higher G+C content in metazoans than in yeast and are therefore more likely to be bound by nucleosomes [20]. As discussed above, such nucleosomes are actively removed in cells where the corresponding promoter is expressed, possibly accounting for the observed discrepancies between predicted and actual nucleosome positioning. Nucleosomes may be repositioned from such G+C rich promoter regions by a variety of mechanisms, including competition with sequence-specific transcription factors [30], [31] or the RNA Polymerase II complex [13], [14], [19], [32], [33], as well as by the action of ATP-dependent nucleosome remodelers (reviewed in [34]). It is also worth noting that regions defined as NDRs are not necessarily completely devoid of nucleosomes [33], [35], but may represent sites with less robust nucleosomes, perhaps because they contain histone variants such as H2.AZ or H3.3 that are less stably bound to DNA [36]. Such nucleosomes are more easily displaced and might therefore make promoters more responsive to inductive signals, but would also make them more sensitive to DNase-based methods used to map nucleosome organization. Taken together, work to date suggests that active processes control nucleosome positioning at many promoters and that this is an important regulatory mechanism for inducible and cell-specific gene expression in metazoans.

Nucleosome organization has been analyzed in blastula stage *O. latipes* (medaka fish [17]) embryos, as well as in samples of mixed stage *D. melanogaster* [13] and *C. elegans* [15], [37] embryos. In spite of metazoan embryos consisting of multiple cell types, these experiments nevertheless detected well-organized nucleosomes. In particular, many promoters reveal a nucleosome arrangement with pronounced nucleosomes flanking the TSS. One nucleosome is observed downstream of the TSS in the coding sequence (+1 nucleosome) and a second upstream of the TSS (−1 nucleosome) with an intervening NDR observed immediately upstream of the TSS. This represents a canonical arrangement in most embryonic cells regardless of tissue type, stage of development or level of transcription. However, it is not clear that such a pattern is truly fixed throughout embryogenesis since chromatin structure appears to be remodeled during embryonic development. For instance, the *hox* genes, which encode homeodomain-containing transcription factors essential for development of all metazoans [38], [39] and that are arranged into several genomic clusters, have been observed to decondense coincident with their expression during mouse embryogenesis [40], [41] – a process that can be mimicked by using retinoic acid (RA; an endogenous inducer of *hox* gene expression) to treat murine ES cells [42]. Chromatin rearrangements at the *hox* clusters have also been observed during mouse embryogenesis using 4C technology [43]. Hence, while the canonical arrangement of a +1 nucleosome at the TSS preceded by an upstream NDR has been observed at *hox* promoters in human cell lines [44], it is unclear if chromatin remodeling during embryonic development generates nucleosome profiles that differ from the canonical organization. Indeed a time course of nucleosome organization, and its refinement in response to inductive signals, has not been reported for any metazoan embryo.

We have mapped nucleosomes near the TSS (herein referred to as ‘promoter’) of 37 zebrafish *hox* genes under different conditions. We first examined nucleosome arrangements at the TSS of all 37 genes at various stages of embryogenesis and find relatively poorly positioned and weakly occupied nucleosomes at 2 hpf and 4 hpf. Notably, no *hox* genes are expressed at these stages of development and we do not observe NDRs at these time points. At the 6 hpf and 9 hpf time points nucleosomes become better organized. The progressive nature of nucleosome positioning on the invariant sequence of *hox* promoters through early development suggests an important role for trans-factors in positioning nucleosomes at *hox* promoters. More detailed analyses revealed that promoters of genes expressed at these stages have better nucleosome organization and occupancy with an NDR immediately upstream of the TSS. Non-expressed promoters have nucleosomes that are less organized and lack an NDR at early stages, suggesting that NDR formation correlates with gene expression. However, blocking *hox* gene transcription by disruption of the RA signaling pathway results in no change in nucleosome positioning or NDR formation, indicating that transcription does not drive nucleosome organization at *hox* promoters. Our data therefore indicate that trans-factors act at *hox* promoters during embryogenesis to dynamically rearrange nucleosomes independently of *hox* gene transcription.

## Materials and Methods

This study was performed in strict accordance with the recommendations in the Guide for the Care and Use of Laboratory Animals of the National Institutes of Health. The protocol was approved by the Committee on the Ethics of Animal Experiments of the University of Massachusetts (A-1565).

### Fish Care

Ekkwill (EK) embryos were collected through natural matings and staged using morphological criteria for two, four, six, and nine hours post fertilization (hpf) as defined by Kimmel et al [45].

### Drug Treatments

Retinoic acid (RA): 2 cell embryos (∼45 minutes post-fertilization) were treated with 100 nM RA diluted in fish-water (5 mM NaCl, 0.17 mM KCL, 0.**33** **mm** CaCl_2_, 0.33 mM MgSO_4_, and 0.004% methylene blue). Embryos remained in RA-treated water until they were harvested (2 hpf RA embryos were treated for ∼1 hour, 4 hpf embryos ∼3 hours etc.). Diethylaminobenzaldehyde (DEAB): 4–8 cell embryos (∼1–1.25 hours post fertilization) were treated with 10 uM DEAB diluted in fish-water. Embryos remained in DEAB-water until the developmental stage harvested. Drug concentrations were chosen based on embryonic survival to limit embryonic death.

### Embryo Processing and Nucleosome Cross-linking

Embryos were collected and the chorion was removed using 10 mg/ml Pronase. Embryos were then washed with Fish ringers (0.1 M NaCl, 3 mM KCl, 3 mM CaCl_2_, 2.4 mM NaHCO_3_) and mechanically dissociated by pipetting. Cells were washed once with PBS, resuspended in 1% formaldehyde in PBS and incubated for 10 minutes at 27°C. The reaction was quenched with equal volume of 1M glycine and cells were spun down at 5000 g.

### Nuclei Purification

Protocol was adapted from Dennis et al 2007 [46]. Cell pellets were resuspended by pipetting vigorously in sucrose buffer (0.3 M sucrose, 2 mM MgAc_2_, 3 mM CaCl_2_, 1% Triton X-100, 500 uM DTT, 1× complete protease inhibitor Roche: 11873580001, and 10 mM HEPES at pH 7.8) and incubated for 30 minutes on ice. Cells were pipetted vigorously again and diluted 1∶1 with GB buffer (25% glycerol, 5 mM MgAc2, 0.1 mM EDTA, 500 uM DTT, 1× complete protease inhibitor Roche: 11873580001, and 10 mM HEPES at pH 7.8). Nuclei were purified by layering on an equal volume of GB and spun at 1000 g for 10 minutes at 4°C.

### MNase Digestion and Chromatin Purification

Protocol was adapted from Yuan et al 2005 [3]. Isolated nuclei were resuspended and washed once in Reaction buffer (50 mM NaCl, 10 mM Tris pH 7.4, 5 mM MgCl_2_, 1 mM CaCl_2_, 1 mM β-mercaptoethanol, 500 uM spermidine and 500 uM DTT) followed by resuspension in reaction buffer with a titrated amount of MNase (5–20 units/ml, Worthington: LS004797) and incubated at 37°C for 10 minutes. Reactions were terminated with 50 mM EDTA and placed on ice. Samples were then diluted in water and treated with 1× RNase cocktail (Ambion: AM2286) and 200 mM NaCl (to remove RNA and reverse crosslinks) and incubated at 55°C for 2 hours. 2 ul proteinase K (20 mg/ml) was added and samples were placed at 65°C overnight. Chromatin was extracted using phenol:chloroform followed by ethanol precipitation. Samples were visualized by gel electrophoresis and samples containing a 80–90% mono-nucleosome DNA (faint tri-nucleosome band visible) were used for tiling array hybridization. Mono-nucleosome sized fragments were gel extracted using the Qiagen Gel Extraction kit (28706).

### Array Build and Hybridization

Zebrafish genome v7 sequence of the seven *hox* clusters was masked for repetitive sequence using the Sanger Institute’s Zebrafish RepeatMasker (http://www.sanger.ac.uk/Projects/D_rerio/fishmask.shtml). The resulting sequences were used to construct a 144 k feature array of 50 bp probes positioned every 20 bp designed using Agilent eArray web software (https://earray.chem.agilent.com/earray/GEO: GPL16536). Isolated mono-nucleosome sized fragments were hybridized to the *hox* array using protocols adapted from Agilent protocols substituting COT DNA for salmon sperm DNA (Mammalian ChIP-on-chip Protocol G4481-90010). Arrays were scanned using either an Axon 4000B or Agilent’s High-Resolution C Scanner.

### Array Analysis and Nucleosome Positioning

Probe sequences were remapped to Zv9 and the distance from the center of a probe to the TSS of the nearest *hox* gene was calculated. Log2 ratios were calculated based on normalized r-processed and g-processed signals from the Agilent chip for each probe. Mean signal from two replicates for each sample was assigned to each probe location. Signals were tallied using a 30 bp sliding window with a step of 10 bp for each window. A Lowess fitting line (f = 0.05) was plotted to show the trend of the aggregated signals. Nucleosome spacing was calculated based on the predicted di- and mono-nucleosome sized fragments identified from gel images, represented in figure S2 in File S1. Our observations indicate that the di-nucleosome band is 320–360 bp, the mono-nucleosome band 150–175 bp and the linker is 20–60 bp, indicating that the peak-to-peak distance between neighboring nucleosomes is 170–210 bp. This distance was used in the text when comparing observed peak distances in the aggregate nucleosome plots. Signals for expressed and non-expressed genes were compared using a two-sided non-paired Wilcoxon rank sum test to calculate the significance of the difference between the two gene sets (GEO: GSE43757 ).

### *hox* Expression

*hox g*ene expression was determined using both Agilent and Affymetrix Zebrafish expression arrays. Only genes found to be expressed by both platforms were included in the RA and WT expression groups. Agilent Arrays: RNA was isolated from retinoic acid treated and untreated WT zebrafish embryos at 2 hpf, 4 hpf, 6 hpf, and 9 hpf embryos using Trizol (Invitrogen#15596-026) following standard procedures. RNA was processed and hybridized to Agilent Zebrafish (V3) Gene Expression Microarrays (G2519F-026437) essentially as outlined in Agilent protocols. Since no *hox* genes are reported to be expressed maternally, the 2 hpf WT embryonic sample was taken to represent baseline and signal above this baseline was taken to represent expression (GEO: GSE43756 ). Affymetrix Arrays: RNA was isolated from retinoic acid treated embryos at 4 hpf, 6 hpf, and 9 hpf while RNA from untreated embryos was collected at 9 hpf. RNA was processed and hybridized to Zebrafish Genechip Arrays (900487) by the UMass Genomic Core facility using standard Affymetrix protocols. CEL files from Affymetrix were normalized using invariantset probe set and background corrected by mas5 using expresso from the R affy package. Present/absent calls were calculated using mas5 call from R affy package with default parameters (GEO: GSE43755).

### QPCR

DEAB-treated embryos were collected at 9 hpf and RNA was extracted using Trizol. cDNA was synthesized using the Superscript III RT First strand cDNA synthesis kit priming with oligo dT (18080-051). *hox* gene cDNA was quantified by QPCR using the Qiagen QuantiFast SYBR Green PCR kit (204054) on an ABI 7300 thermocycler. *hox* expression was normalized to a beta-actin control. Data represents 3 technical replicates.

### Primers

*hoxb1a:* FWD-5′-ACC TAC GCT GAC TTA TCG GCC TCT CAA GG

RVS-5′-CTC AAG TGT GGC AGC AAT CTC CAC ACG

*hoxb7a:* FWD-5′-CCA TCC GAA TCT ACC CAT GGT GAG CGC

RVS-5′-TCT CGA TAC GCC GCC GTC TTG AAA GG

*hoxb1b:* FWD-5′-GGT TCG TTC AGC AAG TAT CAG GTC TCC CC

RVS-5′-TCT CAA GTT CCG TGA GCT GCT TGG TGG

*hoxb5b:* FWD-5′-CCT AAC CCA GGA CCA GTG CAA GAC GG

RVS-5′-CGT TCC GTC AAA CAC AGA GCG TGC G

*hoxb6b:* FWD-5′-AGT GCA AGA CGG ACT GCA CAG AAC AGG

RVS-5′-CGT TCC GTC AAA CAC AGA GCG TGC G

*hoxc8a:* FWD-5′-AGC AAG AGG CCA CCT TAG CGC AAT ACC

RVS-5′-CTT CAA TAC GGC GCT TGC GTG TGA GG

*hoxc9a:* FWD-5′-CGG AGA CTG TTT GGG CTC GAA CGG A

RVS-5′-ACC TCA TAT CGC CGG TCT CTT GTG AGG T

*Beta-Actin:* FWD-5′-ATA CAC AGC CAT GGA TGA GGA AAT CC

RVS-5′-GGT CGT CCA ACA ATG GAG GGG AAA A

### Transcription Start Sites and Genes Included in Study

For this study we used the Embryonic Transcriptome TSSs determined in Pauli et al [47]. Genes with multiple TSSs were left out of this study. This resulted in the inclusion of 37 of the 44 known Zebrafish *hox* genes (Table 1).

**Table 1:** *hox* gene expression during zebrafish embryogenesis.

9 hpf WT non-expressed	9 hf WT expressed	6 hpf RA treated uninduced	6 hpf RA treated induced	RA-only
hoxa4a	hoxb1a	hoxa9a	hoxa4a	hoxa4a
hoxa5a	hoxb7a	hoxa11a	hoxa5a	hoxa5a
hoxa9a	hoxb5b	hoxa13a	hoxb1a	hoxb5a
hoxa11a	hoxb6b	hoxa9b	hoxb5a	hoxc1a
hoxa13a	hoxc8a	hoxa11b	hoxb5b	hoxc4a
hoxa9b	hoxc9a	hoxa13b	hoxb6b	hoxc5a
hoxa11b		hoxb2a	hoxc1a	
hoxa13b		hoxb4a	hoxc4a	
hoxb2a		hoxb6a	hoxc5a	
hoxb4a		hoxb7a		
hoxb5a		hoxb9a		
hoxb6a		hoxb13a		
hoxb9a		hoxb8b		
hoxb13a		hoxc6a		
hoxb8b		hoxc8a		
hoxc1a		hoxc9a		
hoxc4a		hoxc10a		
hoxc5a		hoxc11a		
hoxc6a		hoxc12a		
hoxc10a		hoxc13a		
hoxc11a		hoxc6b		
hoxc12a		hoxc12b		
hoxc13a		hoxd4a		
hoxc6b		hoxd9a		
hoxc12b		hoxd10a		
hoxd4a		hoxd11a		
hoxd9a		hoxd12a		
hoxd10a		hoxd13a		
hoxd11a				
hoxd12a				
hoxd13a				

## Results

To investigate nucleosome organization at *hox* promoters during embryogenesis, we used zebrafish (*Danio rerio)* embryos from 2, 4, 6, and 9 hours post fertilization (hpf). These time points were chosen since zygotic gene expression is initiated at 3–4 hpf in the zebrafish [48]. Hence, 2 hpf and 4 hpf embryos consist of a relatively uniform population of largely undifferentiated cells in which *hox* genes are not transcribed, while in 6 hpf and 9 hpf embryos some cell populations have begun to differentiate and *hox* gene transcription is being initiated. Nucleosome densities were determined by micrococcal nuclease (MNase) digestion of cross-linked chromatin isolated from staged embryos (adapted from [46]). Mononucleosome sized fragments were gel-purified and hybridized to an Agilent custom DNA array tiled with 50 bp oligonucleotides positioned every 20 bp across the seven zebrafish *hox* clusters. Randomly fragmented mononucleosome sized genomic DNA (gDNA) was co-hybridized as a control. The nucleosomal signal was expressed as a ratio of the MNase digested fragments to the random gDNA fragments. Nucleosome densities were averaged for 37 zebrafish *hox* genes (Table 1) from −600 bp to +600bp relative to the annotated transcription start site (TSS). Two separate MNase digestions were carried out for each time point and we find that the results are highly reproducible (r^2^ values range from 0.70 to 0.93; S1 in File S1).

### Nucleosome Organization at *hox* Promoters is Dynamic during Embryogenesis

MNase digests revealed that mononucleosome fragments are 150–175 bp and dinucleosome fragments are 320–360 bp in zebrafish (Fig. S2 in File S1), indicating that linker regions range from 20–60 bp. This is similar to results seen for other fish species [17]. Based on these observations, the expected distance between two nucleosome peaks is 170–210 bp.

**Figure 1:**
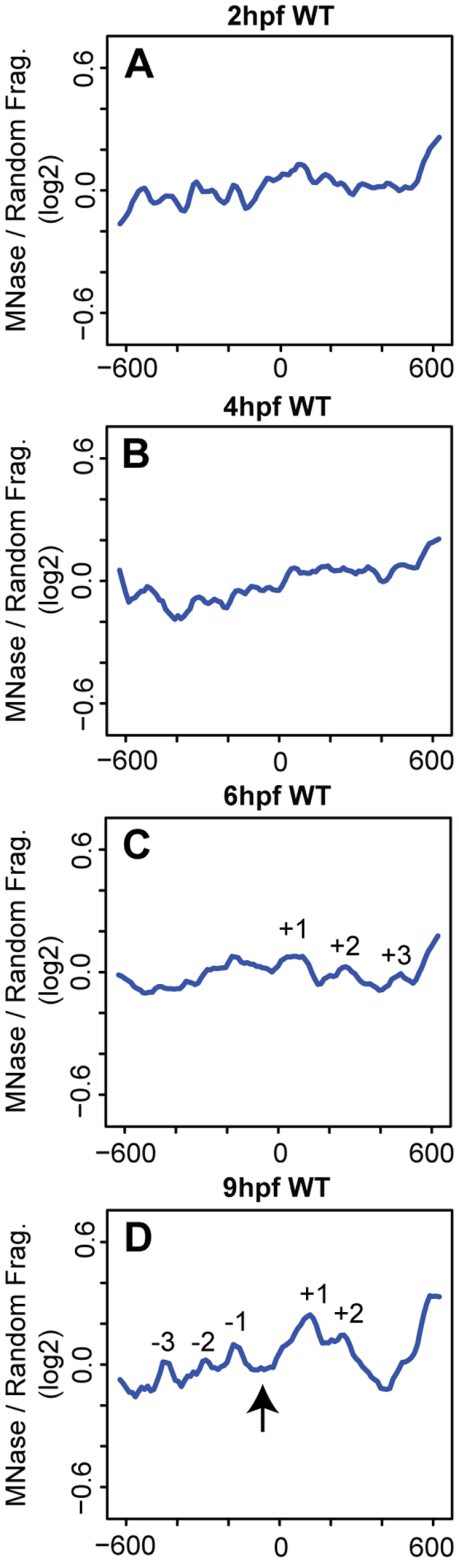
Nucleosome positioning is progressive during early embryonic development. (A–D) Average nucleosome density for 37 zebrafish *hox* promoters was calculated as the log2 ratio of MNase digested to randomly fragmented genomic DNA for positions −600 to +600 relative to the TSS (TSS is set as 0 on X-axis) at 2 hpf (A), 4 hpf (B), 6hpf (C) and 9hpf (D). Detectable nucleosome peaks are numbered in panels C (at positions +60, +260 and +480, separated by 200 bp and 220 bp respectively) and D (at positions −450, −290, −170, +155, and +250 bp, separated by 150 bp, 120 bp, 290 bp, and 130 bp respectively). Arrow in panel D indicates a nucleosome depleted region (NDR) formed between the −1 and +1 nucleosomes.

Our analysis revealed that nucleosomes are poorly occupied and positioned in 2 hpf and 4 hpf embryos (Fig. 1A and B). In particular, we are unable to identify any peaks that correspond to the predicted size of a nucleosome at these stages. Instead peaks have low amplitudes and are broad, indicating low occupancy and a lack of uniform positioning in the promoter region. At 6 hpf, nucleosome peaks begin to appear roughly +60, +260 and +480 bp from the TSS (+1, +2, and +3 nucleosomes respectively in Fig. 1C). The spacing of these peaks (200 bp and 220 bp respectively) indicates a nucleosomal unit of ∼150 bp of protected sequence separated by a linker fragment of ∼60 bp – values that correspond to those expected based on our gel analysis. We note that the amplitudes of the peaks in this region remain modest at 6 hpf, suggesting either that nucleosome occupancy is limited in all embryonic cells, or that nucleosomes are becoming more highly occupied in only a subset of cells. As in 2 hpf and 4 hpf embryos, nucleosomes upstream of the TSS are loosely positioned in 6 hpf embryos. At 9 hpf, nucleosome peaks are observed at roughly −450, −290, −170, +115, and +250 bp (−3, −2, −1, +1, and +2 nucleosomes respectively in Fig. 1D). The amplitude of the nucleosome peaks is greater at 9 hpf than 6 hpf. In particular, the amplitude of the +1 peak increases relative to the other peaks, indicating that nucleosome occupancy increases at this position. We interpret the change in nucleosome occupancy and positioning from 6 hpf to 9 hpf to mean that nucleosomes are less uniformly positioned at 6 hpf and take on more uniform positions by 9 hpf. However, the distances between the −3/−2, −2/−1 and +1/+2 peaks (150 bp, 120 bp, and 130 bp respectively) are closer than the expected distance between nucleosomes, possibly due to nucleosomes occupying different positions between expressed and non-expressed genes, as explored further below. Our results suggest that the arrangement of nucleosomes at *hox* promoters is established gradually during zebrafish embryogenesis.

Several groups have reported a nucleosome-depleted region (NDR) flanked by −1 and +1 nucleosomes upstream of the TSS in many metazoan genes (including *hox* genes) regardless of their expression state [12]–[19], [44]. In many of these reports, the size of the NDR corresponds to approximately one nucleosome. At 2 hpf, 4 hpf, and 6 hpf, nucleosomes around the TSS are too disordered to observe an NDR structure, but we observe an NDR at 9 hpf, where the +1 and −1 nucleosome peaks sit ∼290 bp apart (arrow in Fig. 1D). This is equivalent to an NDR of ∼130 bp, slightly shorter than one nucleosome length. There is also reduced nucleosome density around +400 bp at 9 hpf (Fig. 1D), but the significance of this observation is unclear. Hence, our data indicate that an NDR slightly shorter than one nucleosome is present at 9 hpf.

### Expressed and Non-expressed Promoters Display Distinct Nucleosome Profiles

**Figure 2:**
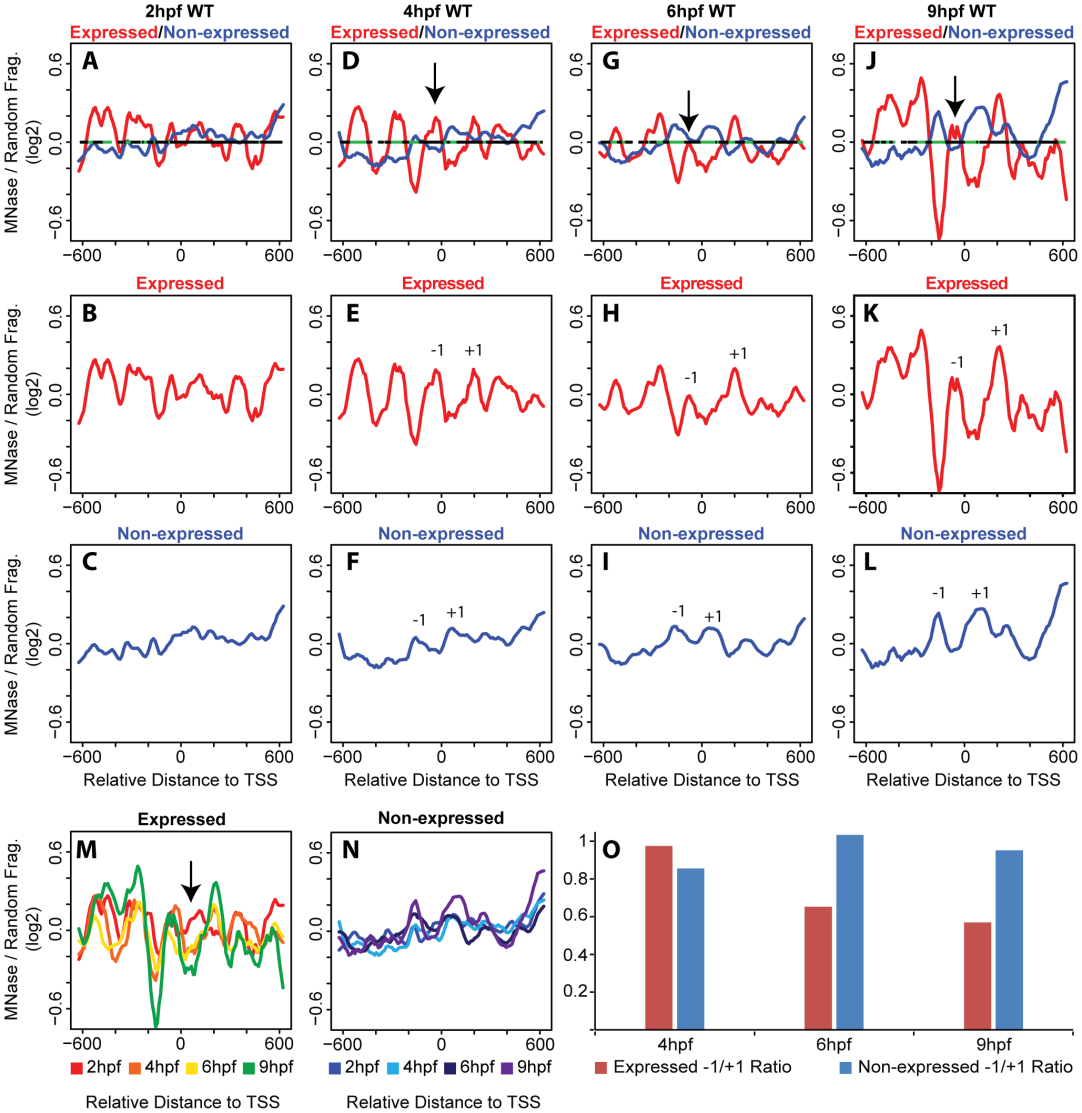
Nucleosome organization differs between expressed and non-expressed promoters. (A–L) Average nucleosome density was calculated as in figure 1 for expressed (red line in panels A, B, D, E, G, H, J, K) and non-expressed (blue lines in panels A, C, D, F, G, I, J, L) promoters at 2 hpf (A–C), 4 hpf (D–F), 6 hpf (G–I) and 9 hpf (J−L). Nucleosome densities at expressed and non-expressed promoters were compared using a Wilcoxon Ranked Sum test and statistically significant differences (p<0.05) are illustrated in green on the horizontal line in panels A, D, G, J. Arrows in D, G, and J indicate the −1 nucleosome. (M, N) Overlay of profiles for expressed (M) and non-expressed (N) promoters at all time points. Arrow in M indicates region where 2 hpf time point (red line) has greater nucleosome density than later time points. (O) Change in occupancy of the −1 nucleosome was calculated as a ratio of density at the −1 nucleosome to density at the +1 nucleosome for expressed (red bars) and non-expressed (blue bars) promoters at 4 hpf, 6 hpf and 9 hpf.

We note that *hox* gene expression is initiated by the 6 hpf and 9 hpf time points, raising the possibility that nucleosome arrangements may be distinct at promoters of transcribed genes relative to promoters of genes which are not transcribed at these stages. To examine this possibility, we first used microarray analysis to identify all *hox* genes that become expressed during the stages analyzed here and find that six *hox* genes are transcribed by 9 hpf (Table 1). We next examined the nucleosome arrangement surrounding the TSS of the 31 non-expressed genes compared to the six genes expressed at 9 hpf. At 2 hpf, promoters of non-expressed genes do not reveal readily apparent nucleosomes (Fig. 2C). However, nucleosomes become progressively more apparent at non-expressed promoters as embryogenesis progresses (Fig. 2F, I) and by 9hpf several well-positioned and well-occupied nucleosomes are detected (Fig. 2L). We note that while there are clear differences in amplitude, nucleosome positioning remains relatively constant across the stages analyzed (Fig. 2N). Since 31 of 37 promoters belong to the non-expressed group, it is expected that the nucleosome profile at non-expressed promoters will closely parallel the profile seen when all promoters are averaged together. While this is indeed the case (compare Fig. 2C, F, I, L to Fig. 1A–D), it is noteworthy that there are also clear differences. For instance, nucleosomes can be seen surrounding the TSS at 4 hpf at non-expressed promoters (−1 and +1 in Fig. 2F), but such nucleosomes are not observed at 4 hpf when all promoters are averaged (Fig. 1B). Furthermore, the −1 nucleosome is better occupied in non-expressed promoters at 9 hpf (Fig. 2L) than when all promoters are averaged (Fig. 1D). These observations suggest that although the number of expressed promoters is small, they must have a distinct nucleosome profile from non-expressed promoters. This turns out to be the case, as can be seen in Fig. 2B, E, H, K. Indeed, promoters of expressed genes reveal relatively well-defined nucleosomes already at 2 hpf (Fig. 2B) and these are further refined by 4 hpf (Fig. 2E), and remain as such at 6 hpf (Fig. 2H) and 9 hpf (Fig. 2K). In addition to being detected earlier than nucleosome peaks at non-expressed promoters, peaks at expressed promoters are also narrower and have higher amplitudes, suggesting that nucleosomes are better positioned and more highly occupied at expressed promoters. As noted for non-expressed promoters, nucleosome positioning also remains relatively constant at expressed promoters across the stages analyzed here (Fig. 2M). One exception is at 2hpf, when nucleosome density is higher near the TSS than at later stages (arrow in Fig. 2M), perhaps indicating that nucleosomes are evicted or repositioned from the TSS upon initiation of gene activation.

A closer examination reveals additional differences in nucleosome positioning at promoters of expressed versus non-expressed *hox* genes. These differences are observed most readily when the profiles are overlayed as in figures 2 A, D, G and J. In particular, in the region surrounding the TSS (−300 to +300), non-expressed promoters display peaks at −160 and +70, while expressed promoters display peaks at −270,−50 and +200. Notably, the −1 nucleosome in expressed promoters (arrow in Fig. 2 D, G, J) appears to be dynamic, as it is reduced at 6 hpf and 9 hpf (when *hox* genes are expressed) relative to 4 hpf (when *hox* genes are not expressed). This is particularly clear when the amplitude of the −1 nucleosome peak is compared to the amplitudes of the adjacent peaks. Expressing the amplitude of the −1 nucleosomes as a ratio to the +1 nucleosomes reveals that the −1 nucleosome in expressed promoters at 6 hpf and 9 hpf is reduced by 35% and 43%,respectively (Fig. 2O), while the −1 nucleosome remains unchanged in the non-expressed promoters. The net result is a reduction in nucleosome density between the −270 and +200 peaks in the expressed promoters at stages when *hox* genes are expressed. While this is consistent with previous reports of NDRs forming at expressed promoters, the region is not devoid of nucleosomes since a peak persists at the TSS at 6 hpf and 9 hpf. It is possible that this peak represents a less stable nucleosome or that it reflects the fact that not all cells in the embryo express these *hox* genes, but our experiments cannot distinguish between these possibilities.

In an attempt to determine the significance of the observed differences between expressed and non-expressed promoters, we employed a two-sided Wilcoxon rank sum test. The results of this test are indicated on the horizontal line in figure 2A, D, G, J where regions with a statistically significant difference in nucleosome density between expressed and non-expressed promoters are indicated in green. As can be seen, the greatest difference between the two conditions is centered near the TSS at 6 hpf and 9 hpf, although other regions (most notably the region −200 to −600 in 4 hpf embryos) also show significant differences. We conclude that nucleosomes are detectable earlier at promoters of expressed *hox* genes and that these nucleosomes are better positioned and more highly occupied than nucleosomes at promoters of non-expressed *hox* genes. We further conclude that nucleosome occupancy changes as *hox* genes become expressed such that nucleosome density decreases near the TSS, although we do not observe the formation of a region truly depleted of nucleosomes. Hence, *hox* promoters may fall into the class of promoters where a nucleosome positioned upstream of the TSS must be actively removed prior to transcription, thereby providing additional regulation and permitting high transcriptional plasticity.

### Disruption of Retinoic Acid Signaling Blocks *hox* Transcription, but does not Affect Nucleosome Organization

**Figure 3:**
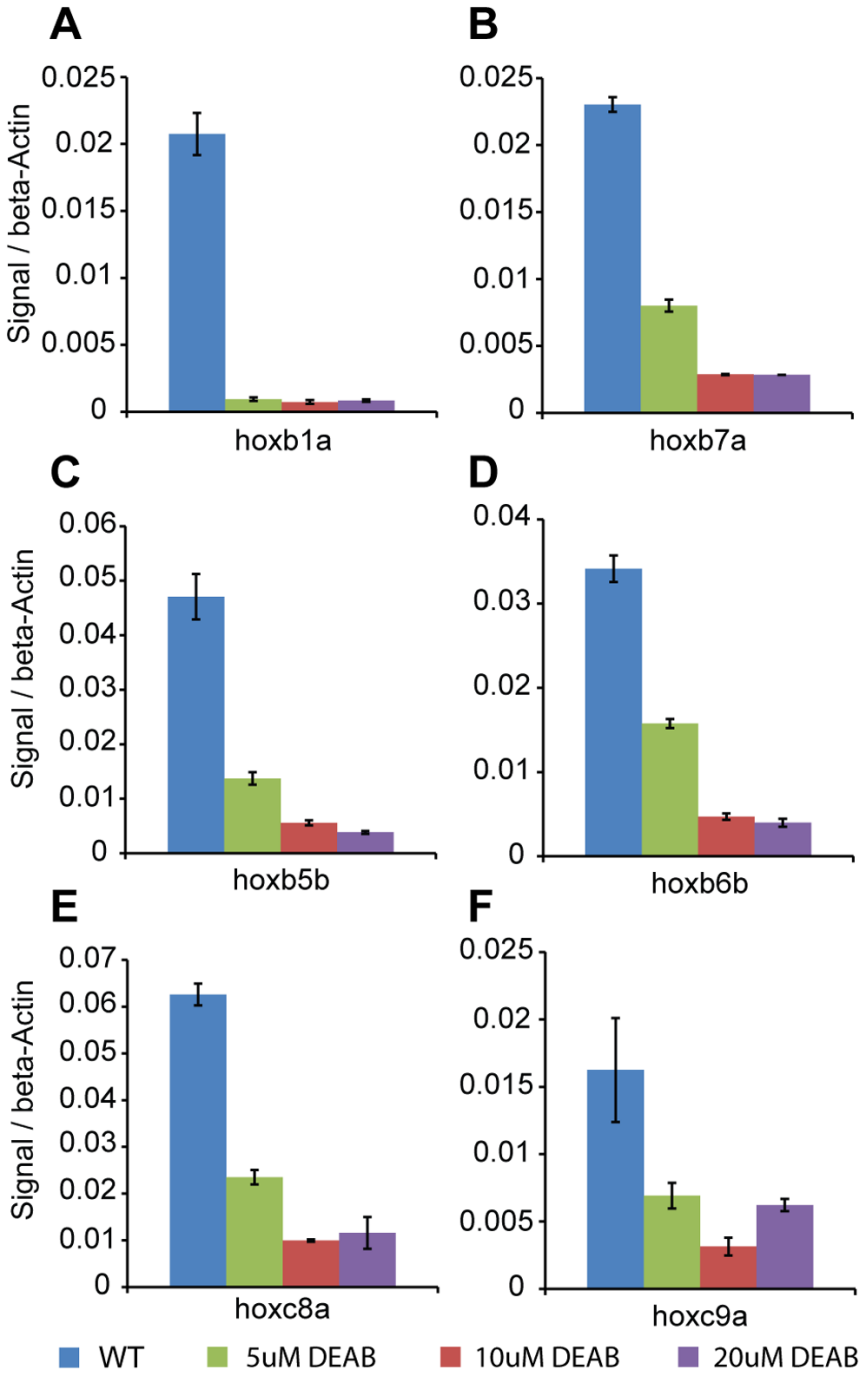
DEAB treatment blocks *hox* transcription. (A–F) Zebrafish embryos were left untreated (blue bars) or treated with 5 uM (green bars), 10 uM (red bars) or 20 uM (purple bars) DEAB and harvested at 9 hpf. Transcript levels for *hoxb1a* (A), *hoxb7a* (B), *hoxb5b* (C), *hoxb6b* (D), *hoxc8a* (E) and *hoxc9a* (F) were determined by quantitative RT-PCR and normalized to β-actin. Error bars indicate standard deviations of 3 technical replicates.

**Figure 4:**
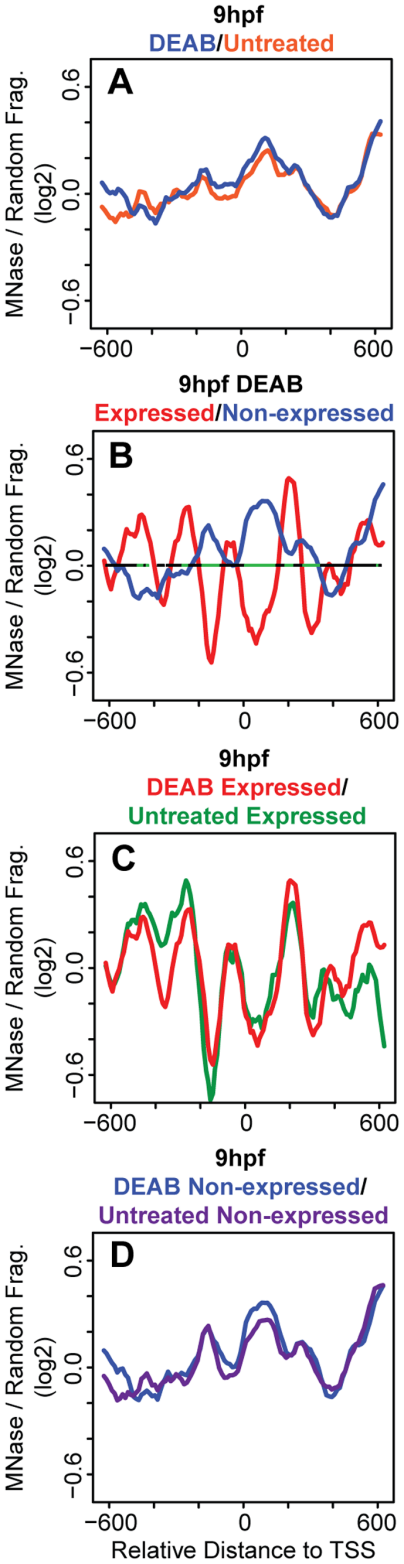
DEAB treatment has little effect on nucleosome organization at *hox* promoters. (A–D) Average nucleosome density was calculated as in figure 1. (A) Overlay of average nucleosome profiles for 37 *hox* promoters from DEAB-treated (blue line) and untreated (orange line) embryos at 9 hpf. (B) Overlay of nucleosome profiles for expressed (red line) and non-expressed (blue line) promoters in DEAB-treated embryos at 9 hpf. Nucleosome densities at expressed and non-expressed promoters were compared using a Wilcoxon Ranked Sum test and statistically significant differences (p<0.05) are illustrated in green on the horizontal line in panel B. (C) Overlay of nucleosome profiles for expressed promoters from DEAB-treated (red line) and untreated (green line) embryos at 9 hpf. (D) Overlay of nucleosome profiles for non-expressed promoters from DEAB-treated (blue line) and untreated (purple line) embryos at 9 hpf.

As mentioned, the retinoic acid (RA) signaling pathway is an activator of *hox* gene expression and plays a role in chromatin rearrangements at the *hox* clusters in both cell lines and mouse embryos [40]–[42]. To test if the RA signaling pathway plays a role in the nucleosome positioning observed in our experiments, we treated embryos with diethylaminobenzaldehyde (DEAB), a compound that blocks the RA synthesis pathway by inhibiting retinaldehyde dehydrogenase (RALDH) [49]. DEAB has also previously been shown to affect hindbrain development, particularly *hox* gene expression, in zebrafish embryos [50]. DEAB treatment was begun at the 2–4 cell stage in order to prevent initiation of *hox* transcription and embryos were collected at 9 hpf to determine transcript levels and nucleosome organization of the six active *hox* genes. RT-qPCR analysis revealed that transcription of the six *hox* genes was maximally blocked by 10 uM DEAB, with higher DEAB concentrations not providing further blockade (Fig. 3). Plotting average nucleosome profiles for all 37 *hox* genes from DEAB-treated embryos revealed no change from untreated embryos (Fig. 4 A). When *hox* genes are divided into expressed and non-expressed groups, nucleosomes in DEAB-treated embryos are again positioned very similarly to untreated embryos (Fig. 4B, compare to Fig. 2J). Overlaying nucleosome traces for expressed and non-expressed genes from DEAB and untreated embryos confirms the similarity (Fig. 4C, D). Hence, while the six genes expressed at these stages are RA sensitive and blocking RA synthesis disrupts their transcription, no detectable change in nucleosome organization is observed in the absence of RA signaling. We conclude that RA-induced transcription is not driving changes in nucleosome organization at the promoter regions of *hox* genes during zebrafish embryogenesis.

### Retinoic Acid Treatment does not Affect Nucleosome Organization at *hox* Promoters

**Figure 5:**
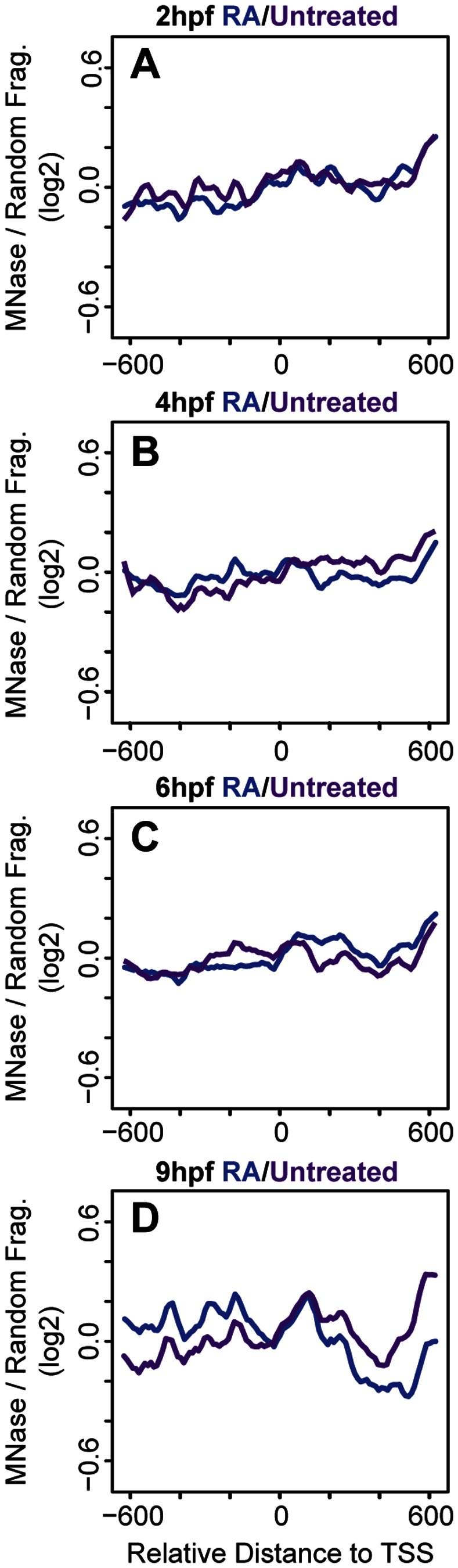
Exogenous RA has little effect on nucleosome organization at *hox* promoters. (A–D) Average nucleosome density was calculated as in figure 1 for 37 *hox* promoters from RA-treated embryos. Overlay of nucleosome profiles for 37 *hox* promoters from RA-treated (blue line) and untreated (purple line) embryos at 2 hpf (A), 4 hpf (B), 6 hpf (C) and 9 hpf (D).

**Figure 6:**
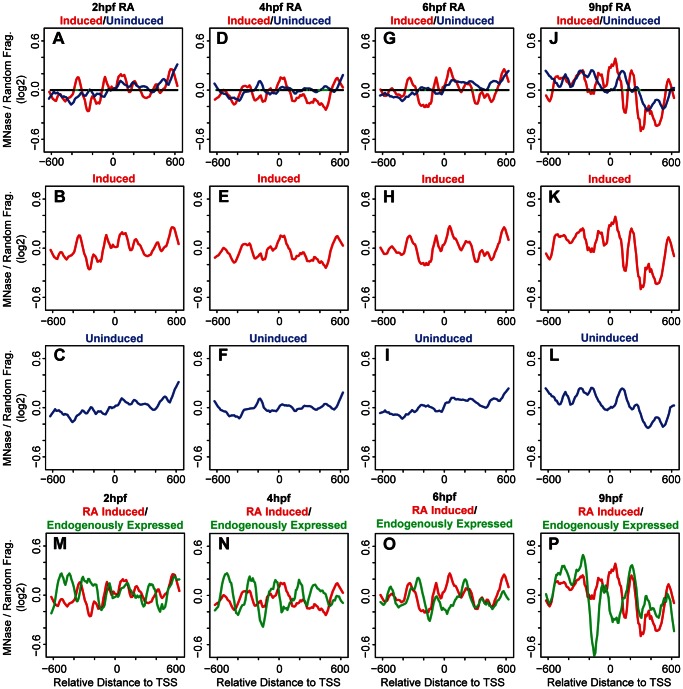
Exogenous RA does not affect nucleosome organization at *hox* promoters. (A–L) Average nucleosome density was calculated as in figure 1 for expressed (red line in panels A, B, D, E, G, H, J, K) and non-expressed (blue lines in panels A, C, D, F, G, I, J, L) promoters at 2 hpf (A–C), 4 hpf (D–F), 6 hpf (G–I) and 9 hpf (J–L). Nucleosome densities at induced and uninduced promoters were compared using a Wilcoxon Ranked Sum test and statistically significant differences (p<0.05) are indicated in green on the horizontal line in panels A, D, G and J. (M–P) Overlay of nucleosome profiles for expressed promoters in untreated (green line) and RA-treated (red line) embryos at 2 hpf (M), 4 hpf (N), 6 hpf (O) and 9 hpf (P).

We next examined if addition of exogenous RA affects nucleosome organization at *hox* promoters. Embryos were treated with RA starting at the 2-cell stage and collected at 2 hpf, 4 hpf, 6 hpf and 9 hpf. We initially examined average nucleosome organization at all 37 *hox* promoters. We find the nucleosome profiles of RA-treated embryos to be similar to the profiles of untreated embryos, although there are some minor differences when overlayed (Fig. 5A–D). Using microarray analysis we identified nine *hox* genes whose expression is induced in RA treated embryos (Table 1). We next used this information to compare nucleosome organization at RA-induced and uninduced *hox* promoters. Promoters of genes not induced by RA do not display detectable nucleosomes until 9 hpf (Fig. 6C, F, I, L). As expected, this is similar to the non-expressed promoters in untreated embryos (Fig. 2C, F, I, L), although it is somewhat more difficult to detect individual nucleosomes in RA treated embryos and there may be additional nucleosomes forming in the region of −200 to −600 at 9 hpf (Fig. 6L). RA-induced promoters (Fig. 6B, E, H, K) show better positioned and more highly occupied nucleosomes than uninduced promoters (Fig. 6C, F, I, L) as can be seen when profiles of the two groups are overlayed (Fig. 6A, D, G, J). However, there are essentially no regions with statistically significant differences between RA-induced and uninduced promoters. This finding is in contrast to the changes in nucleosome organization we observed when comparing expressed and unexpressed promoters in untreated embryos (Fig. 2A, D, G, J) and suggests that although RA induces transcription of several *hox* genes, it does not drive their nucleosome organization to mimic that of endogenously expressed genes. Indeed, when the nucleosome profiles of RA-induced promoters (from Fig. 6B, E, H, K) are overlayed on the profile of endogenously expressed promoters (from Fig. 2B, E, H, K) it is clear that the profiles differ (Fig. 6 M–P). In particular, while nucleosomes are depleted in the region from −100 to −200 in both sets of promoters at 4, 6, and 9 hpf, this depletion is less pronounced at RA-induced promoters and depletion in the region from 0 to +100 is not observed at all at RA-induced promoters.

**Figure 7:**
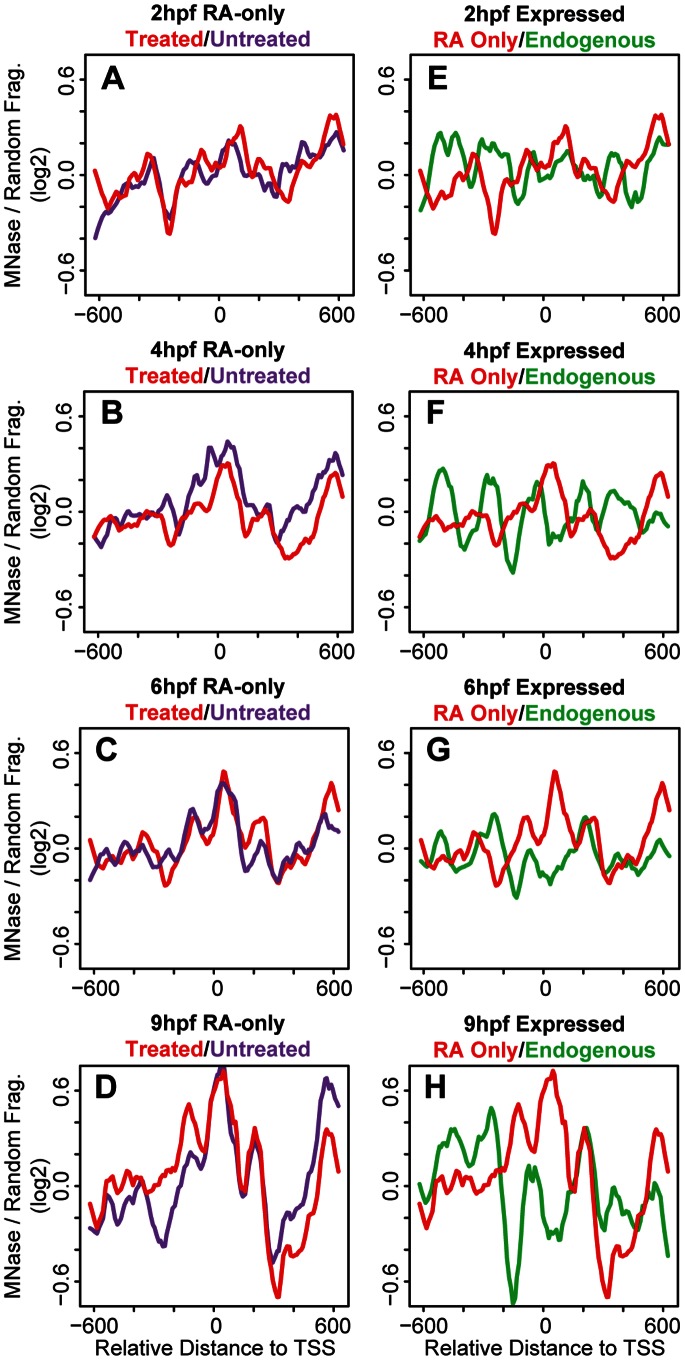
Exogenous RA does not affect nucleosome positioning at induced promoters. (A–H) Average nucleosome density was calculated as in figure 1 for RA-only genes in RA-treated embryos (red line in panels A–H), for RA-only genes in untreated embryos (purple line in panels A–D), and for endogenously expressed genes in untreated embryos (green line in panels E–H).

We note that three *hox* genes are shared between the group of endogenously expressed genes and the group of RA-induced genes (Table 1). To better isolate the effects of RA, we created a third group of promoters that are only induced by RA (Table 1; RA-only). Overlays of the nucleosome profiles of the six RA-only promoters from RA-treated embryos on the profiles of the same promoters from untreated embryos, reveal the nucleosome profiles to be similar (Fig. 7A–D). Hence, while RA induces the expression of these six *hox* genes, it has no effect on nucleosome organization at their promoters. Furthermore, the nucleosome organization at induced RA-only promoters is clearly distinct from that of endogenously expressed promoters (Fig. 7 E–H). Taken together, the results of our DEAB and RA treatments demonstrate that RA regulates *hox* gene transcription, but does not drive nucleosome organization at *hox* promoters during early zebrafish development.

## Discussion

While nucleosomes have been mapped in several different systems, little is known about nucleosome organization in a developing vertebrate embryo. Initial analyses of nucleosome organization focused on yeast and cultured cells that represent relatively uniform populations and that, while responsive to some stimuli, in many cases have relatively limited developmental potential. In contrast, developing embryos are multicellular and contain diverse cell types that represent a range of developmental potentials. Recent studies have analyzed nucleosome arrangements in *C. elegans* [15], [35] and *D. melanogaster* [13] embryos using mixtures of embryonic stages. However, this strategy limits the ability to detect changes in chromatin structure at specific developmental stages. Here we use staged zebrafish embryos to analyze the nucleosome arrangement at *hox* promoters during vertebrate embryogenesis. We find that nucleosomes are poorly organized at early stages, but become better organized by 6 hpf and 9 hpf. These latter stages correspond to the time when *hox* genes first become expressed in the embryo. Comparing expressed and non-expressed genes, we observe several differences in nucleosome organization at the promoter regions. First, we observe increased nucleosome occupancy at expressed promoters when compared to non-expressed promoters. Interestingly, the increased amplitude is observed in most of the nucleosomes in the promoter region, with exception of the −1 nucleosome. We find that occupancy of the −1 nucleosome decreases at 6 hpf and 9 hpf at expressed promoters. Second, we detect changes in the spacing between the −1 and +1 nucleosomes of expressed and non-expressed promoters. The larger spacing is most evident at 6 hpf and 9 hpf in the expressed promoters and coincides with a likely NDR. Due to this change in spacing, nucleosomes also appear out of phase between the expressed and non-expressed promoters. Finally, though *hox* transcription is dependent on RA signaling, we find that blocking RA signaling does not cause changes in nucleosome organization at the expressed promoters, suggesting that nucleosome arrangement is independent of RA-induced transcription. The fact that nucleosome organization is dynamic, but genomic sequence is invariant, during embryogenesis, also suggests that trans-factors play a role in dynamically positioning nucleosomes at the promoters of *hox* genes in the developing embryo.

### The Role of Transcription in Nucleosome Organization at *hox* Promoters

Transcription has been shown previously to correlate with specific nucleosome profiles at some TSSs in metazoans [12], [18], [19]. Indeed, in our bulk nucleosome plots at 9 hpf, when *hox* transcription is initiated, nucleosomes appear to be better positioned as compared to bulk nucleosome positions at 2 hpf−6 hpf (Fig. 1A–D). Grouping the *hox* genes into expressed and non-expressed promoters revealed that nucleosomes at expressed promoters are better positioned and have increased occupancy when compared to nucleosomes at non-expressed promoters (Fig. 2A, D, G, J). While these data suggest that transcription may have a direct effect on the nucleosome arrangement at *hox* promoters, we find that blocking RA signaling represses *hox* transcription (Fig. 3) with no changes in the nucleosome profile (Fig.4). We note that our DEAB protocol was designed to prevent initiation of *hox* transcription and that we may have observed a different effect if *hox* gene transcription had been allowed to initiate prior to being inactivated. Hence, our data suggest that the nucleosome profile at *hox* promoters is independent of RA-induced *hox* transcription. We see further support for this conclusion when embryos are treated with RA. Though exogenous RA induces *hox* transcription, RA-induced genes do not recapitulate the nucleosome positions observed at endogenously expressed promoters (Fig. 6) and display little change from nucleosome positions observed in untreated embryos (Fig. 7), again suggesting that the nucleosome profile at *hox* promoters is independent of *hox* transcription.

Our findings raise the question as to what role RA signaling plays in *hox* transcription if it does not affect nucleosome organization. Given the complexity of eukaryotic chromatin structure, it is possible that RA affects chromatin structure at a level distinct from the nucleosome. For instance, previous studies detected chromatin changes at the *HoxB* and *HoxD* clusters using fluorescent in situ hybridization [40]–[42]. *Hox* loci were observed to decondense during mouse embryogenesis in correlation with *hox* gene transcription and this process was recapitulated by RA-treatment of ES cells. It is therefore possible that RA affects chromatin at the level of the 30 nm fiber without affecting the positioning of individual nucleosomes. It is also possible that RA affects *hox* expression by promoting histone modifications that are supportive of transcription. Indeed, RA receptors are known to recruit histone-modifying enzymes [51]. Lastly, RA may simply recruit components of the transcription machinery, again via RA receptors, to *hox* promoters. The fact that RA induces *hox* transcription without affecting nucleosome organization could also be taken to indicate that many nucleosome arrangements are permissive for transcription. However, it is important to note that the exogenously applied RA is likely in significant excess relative to endogenous levels and this may permit over-riding of a nucleosome arrangement that would not otherwise support transcription. In summary, we propose that an RA-independent mechanism promotes a nucleosome arrangement that is permissive for transcription, but that RA is required for actual transcription. A transcription-independent mechanism for nucleosome organization is also supported by our observation that an NDR forms at non-expressed promoters by 9 hpf. Since genes in this group will become expressed at later stages of embryogenesis, it is possible that this NDR forms in preparation for subsequent transcriptional activation.

### A Likely Role for Trans-factors in Nucleosome Organization at *hox* Promoters

Nucleosome positioning has been shown to result from the combination of intrinsic characteristics of DNA sequence, such as base pair composition (cis-elements), and from factors that interact with DNA, such as transcription factors and ATP-dependent chromatin modifiers (trans-factors). However, the relative contribution of each mechanism remains unclear. A recent study addressed how cis-elements and trans-factors influence nucleosome positioning in yeast. By using YACs to transfer large DNA fragments between divergent yeast strains, analysis of nucleosome organization in the native strain could be compared to nucleosome organization on the YAC in the new host strain [52]. This analysis revealed that inter-nucleosome spacing and positioning of the +1 nucleosome was altered upon transfer to the new host strain. Since sequence remains constant between the YAC and native yeast strain, these findings suggest that trans-factors play a more important role in nucleosome positioning than cis-elements. Similarly, we find that nucleosome organization changes during embryogenesis, but since the underlying sequence is invariant during development, trans-factors also likely play a role in nucleosome positioning during embryogenesis. We note that the changes in nucleosome organization that we observe correlate with important transitions during embryonic development. In particular, 2 hpf and 4 hpf embryos display relatively disordered nucleosomes at promoter regions (Fig. 1A, B), while at 6 hpf and 9 hpf nucleosomes are readily identified (Fig. 1C, D). This change coincides with activation of the zygotic genome at the maternal zygotic transition (MZT), which occurs in a time window at approximately 3–4 hpf. Our data do not reveal whether there is a causal relationship between this transition and the observed nucleosome rearrangement. However, since we observe better nucleosome positioning after the MZT, it is plausible that trans-factors (such as transcription factors and ATP-dependent chromatin remodelers) become expressed at the MZT and subsequently regulate nucleosome arrangements at *hox* promoters.

### NDR Formation at *hox* Promoters during Embryogenesis

Nucleosome depleted regions (NDRs) were initially identified at promoters in yeast, but have subsequently been identified in other cell types. In most cases, NDRs are readily observed in bulk analyses of promoters regardless of whether the promoters are active or not. Indeed, previous analyses of bulk *hox* promoters in human cell lines identified an NDR upstream of the TSS [44]. Accordingly, when we average nucleosome positions for all 37 zebrafish *hox* genes, we observe an NDR as soon as −1 and +1 nucleosomes are resolved at the TSS (9 hpf, Fig. 1D). The NDR observed in the bulk plot at 9 hpf is ∼130 bp, while the NDRs observed at expressed promoters at 6 hpf and 9 hpf are ∼100 bp and ∼110 bp respectively and the NDR observed at non-expressed promoters at 9 hpf is ∼85 bp, suggesting an average NDR size of ∼100 bp. This is relatively similar to NDRs observed in other genome-wide nucleosome mapping studies, including fish, where NDR lengths vary somewhat, but are ∼150 bp.

Though the NDRs observed in our study are similar to other bulk studies, they are smaller than the NDR previously observed at human *hox* promoters, which was reported to be ∼500 bp [44]. We suspect the difference in NDR lengths between the two studies is due to differences between zebrafish embryos and human cell lines. First, the embryo is made up of a heterogeneous population of cell types, while cell lines represent a homogeneous population. The heterogeneity of cell types in the embryo might lead to variable nucleosome occupancy. For instance, cells in the embryo that do not express a given *hox* gene might have a nucleosome positioned upstream of the TSS, thereby reducing the size of the NDR observed when signals from all cells in the embryo are averaged. Indeed, a previous study found nucleosomes to be differentially positioned at the serum albumin enhancer in a tissue specific manner in mouse [53]. Such variable nucleosome occupancy presumably does not occur in cell lines since they represent a homogeneous population of cells that would all have similar nucleosome positions. Interestingly, if some cells in the embryo lacked the −1 nucleosome, then the NDR of these promoters would expand to 310 bp and 320 bp at 6 hpf and 9 hpf respectively, making it more similar to the NDR observed at *hox* promoters in human cell lines. Second, the difference in NDR length may be due to differences between fish and humans. For instance, divergence of regulatory sequences in the promoters as well as divergence in the trans-factors responsible for nucleosome positioning may lead to different sized NDRs. Support for this possibility comes from the analysis of NDRs in evolutionary divergent yeast species, which were found to have different sized NDRs at orthologous promoters [52].

Our data do not address how NDRs form, but we consider several possibilities. First, NDRs could form in a competitive process. Evidence exists for competition between nucleosomes and trans-factors for binding to specific sequences [54], [55]. Once a trans-factor is bound, positioning of nucleosomes would be restricted to other available sites in a process similar to that suggested by the “barrier model”. The barrier model is driven by trans-factors interacting with DNA and providing a barrier that blocks free nucleosome diffusion, creating well-ordered and positioned nucleosomes [6], [56]. Hence, binding of trans-factors at expressed *hox* promoters would create more uniform nucleosome positions as well as increased amplitude of nucleosome peaks, while the lack of trans-factor binding at non-expressed genes would lead to lower occupancy and less well-positioned nucleosomes. Such competition has been observed at the CLN2 promoter in yeast where binding sites for three sequence specific transcription factors are needed for NDR formation. In the absence of these binding sites, the CLN2 promoter has increased nucleosome occupancy [54]. Meis and Pbx proteins, which bind elements in many *hox* promoters and are involved in the regulation of *hox* transcription, have been suggested to act as pioneer transcription factors capable of binding nucleosome-occupied DNA [57] and may impact nucleosome binding at *hox* promoters. Since RA-receptors may be bound to DNA even in the absence of RA-signaling [58], [59], RARs may play a similar role by binding RA response elements. However, our analyses have failed to identify an enrichment of binding sites for any known trans-factor in the NDR regions of *hox* promoters. Second, NDR formation could be an active process mediated throughout embryogenesis by ATP-dependent remodelers. ATP-dependent SWI2/SNF2 complexes, which slide nucleosomes through DNA sequence, have been previously shown to regulate *hox* genes [60], [61]. Many of these factors do not bind DNA directly and would therefore need to be recruited to *hox* promoters by DNA binding factors such as the Meis and Pbx factors mentioned above.

### Nucleosome Occupancy and Histone Modifications at *hox* Promoters are Temporally Coincident

The accessibility of genomic DNA is regulated not only by nucleosome positioning, but also by post-translational modifications made to the N-termini of histone tails, that in turn affect chromatin structure. For instance, histone H3 lysine 4 tri-methylation (H3K4me3) by trithorax group proteins and histone H3 lysine 27 tri-methylation (H3K27me3) by polycomb group proteins, associate with active and inactive promoters, respectively [62]. A recent study mapped H3K4me3 and H3K27me3 marks throughout the zebrafish genome at 2.5 hpf (pre-MZT), as well as at 4.5 hpf (post-MZT), and detected chromatin marks only post-MZT [63]. Notably, this coincides with the time point where we first observe well-defined nucleosomes. This temporal coincidence of emerging well-positioned nucleosomes and detectable histone modifications suggests that histones may become modified as soon as they are deposited at a promoter. While the significance of this observation is unclear, it is noteworthy that *hox* promoters are bivalently marked with both H3K4me3 and H3K27me3 at this stage [63]. Bivalency is thought to act as a developmental control, poising developmentally important genes for rapid activation at the appropriate stage of embryogenesis [64]. Indeed, the inability to deposit H3K27me3 marks leads to misregulated *hox* gene expression and homeotic transformations in *Drosophila* [65]. Hence, it is possible that recently deposited nucleosomes at *hox* promoters must be rapidly modified in order to ensure proper regulation of *hox* genes.

## Supporting Information

File S1 **File with Figures S1 and S2. Figure S1** **Comparison of biological replicates used for calculation of nucleosome densities.** Data from two biological replicates were plotted against each other for untreated embryos at 2 hpf (A), 4 hpf (B), 6 hpf (C), 9 hpf (D), as well as for RA-treated embryos at 2 hpf (E), 4 hpf (F), 6 hpf (G), 9 hpf (H) and for DEAB-treated embryos at 9 hpf (I). R^2^ values are indicated in the top right quadrant of each panel. **Figure S2** **Representative MNase digestion.** Cross-linked genomic DNA from 4 hpf embryo was left untreated (lane 2) or treated for 10 minutes at 37°C with serially diluted concentrations of micrococcal nuclease (MNase) increasing from 0.5 units/ml −8 units/ml (lanes 3–6) and separated by agarose gel electrophoresis. Lanes 1 and 7 contain size ladders.
(PDF)
