# Supplementary figures and images for: Dynamic Nucleosome Organization at hox Promoters during Zebrafish Embryogenesis
Source: PLoS One. 2013 May 9;8(5):e63175. doi: 10.1371/journal.pone.0063175 (PMC3650070; doi:10.1371/journal.pone.0063175)

Figure S1.

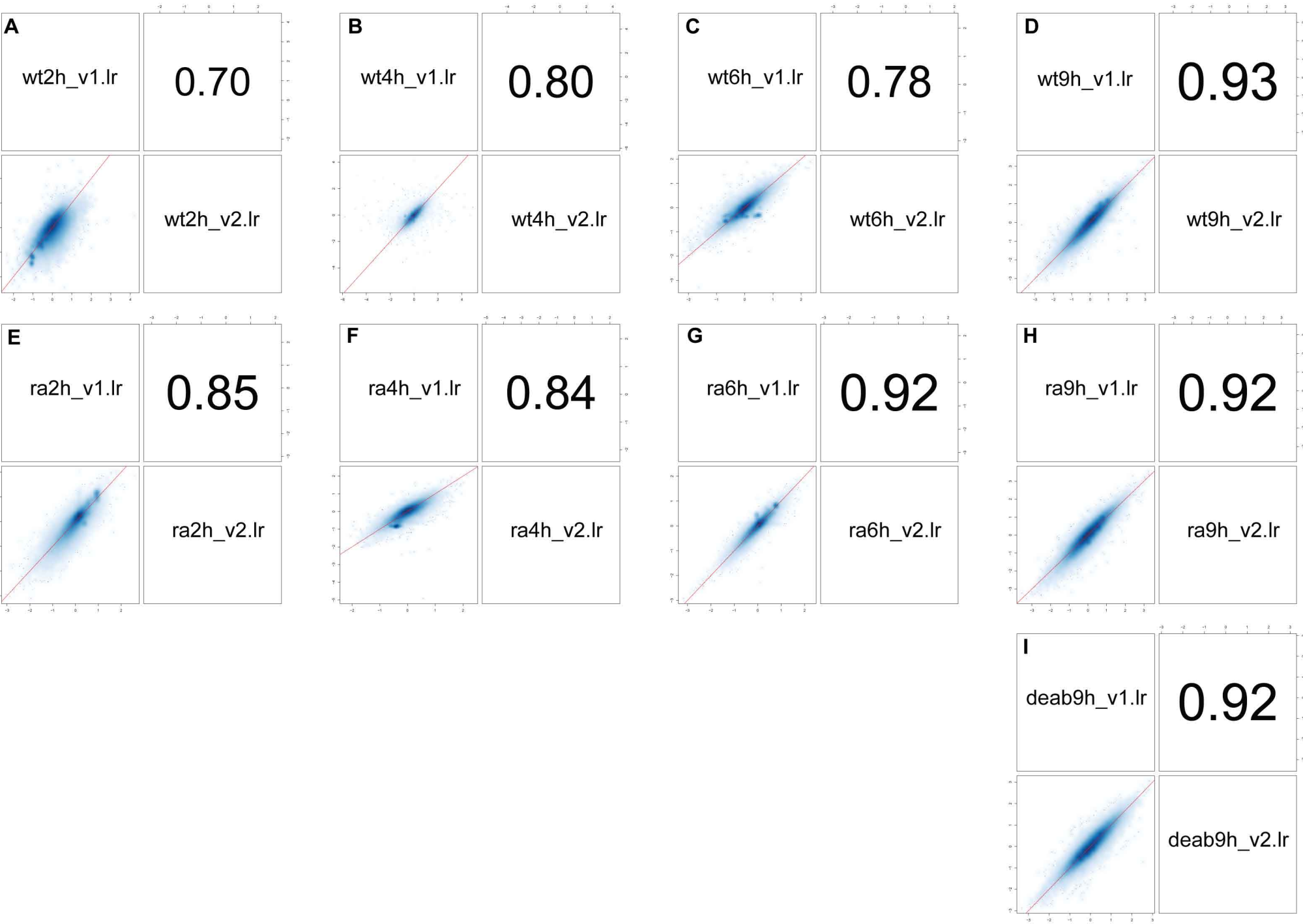

**Figure S2.**

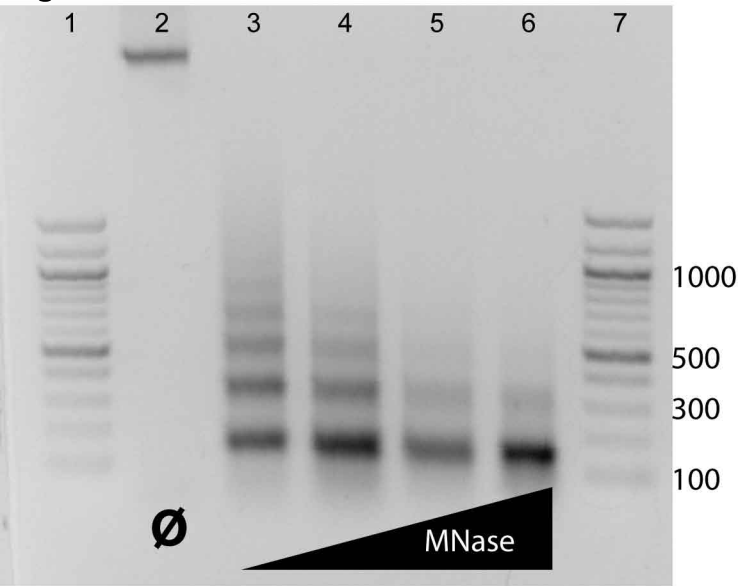

Supplement: File S1 — File with Figures S1 and S2. Figure S1 Comparison of biological replicates used for calculation of nucleosome densities. Data from two biological replicates were plotted against each other for untreated embryos at 2 hpf (A), 4 hpf (B), 6 hpf (C), 9 hpf (D), as well as for RA-treated embryos at 2 hpf (E), 4 hpf (F), 6 hpf (G), 9 hpf (H) and for DEAB-treated embryos at 9 hpf (I). R2 values are indicated in the top right quadrant of each panel. Figure S2 Representative MNase digestion. Cross-linked genomic DNA from 4 hpf embryo was left untreated (lane 2) or treated for 10 minutes at 37°C with serially diluted concentrations of micrococcal nuclease (MNase) increasing from 0.5 units/ml −8 units/ml (lanes 3–6) and separated by agarose gel electrophoresis. Lanes 1 and 7 contain size ladders. (PDF) [file pone.0063175.s001.pdf]
